# The racial and ethnic gap in behavioral measures rivals the gender gap in the United States

**DOI:** 10.1073/pnas.2527671123

**Published:** 2026-02-04

**Authors:** Aurélie Dariel, John C. Ham, Nikos Nikiforakis, Jan Stoop

**Affiliations:** ^a^Center for Behavioral Institutional Design, Research Institute, New York University Abu Dhabi, Abu Dhabi 129188, United Arab Emirates; ^b^Division of Social Science, New York University Abu Dhabi, Abu Dhabi 129188, United Arab Emirates; ^c^New York University Wagner School of Public Service, New York University, New York, NY 10012; ^d^Faculty of Arts and Science, New York University, New York, NY 10003; ^e^Erasmus School of Economics, Erasmus University Rotterdam, Rotterdam 3000 DR, the Netherlands; ^f^Tilburg School of Economics and Management, Tilburg University, Tilburg 5000 LE, The Netherlands

**Keywords:** behavioral measures, competitiveness, risk tolerance, racial/ethnic/gender gaps, United States

## Abstract

Behavioral research has been criticized for relying on demographically narrow WEIRD samples-Western, Educated, Industrialized, Rich, and Democratic. While recent work has expanded to explore cross-country behavioral differences, the study of within-country variation has largely focused on gender, ignoring race and ethnicity—so much so that the “W” in WEIRD might as well stand for “White.” Using incentivized tasks and a large, stratified sample of U.S. adults, we document substantial racial and ethnic gaps in competitiveness and risk tolerance both of which are widely studied behavioral measures: (non-Hispanic) Whites are less competitive and more risk tolerant than Blacks and Hispanics. These gaps are comparable in magnitude to the corresponding gender gaps in our sample. Notably, gender differences do not generalize across racial and ethnic groups: Whereas Whites and Hispanics exhibit gender gaps in competitiveness and risk tolerance, Black women neither shy away from competition nor are they less risk tolerant than Black men. These findings challenge prevailing generalizations in the literature and underscore the importance of examining race and ethnicity in behavioral research.

A well-known critique of behavioral research in the social sciences is that the vast majority of studies rely on WEIRD samples-Western, Educated, Industrialized, Rich, and Democratic-most often composed of university students in North America and Western Europe (1). The limitations of this sampling strategy to the ability of researchers to generalize their findings are well documented: Henrich and coauthors famously argued that WEIRD populations are among the least representative in the world (2). Researchers responded to the call for greater diversity in sampling by examining cross-country variation in behavior (3–5). However, the study of within-country variation remains limited, even though evidence suggests it is as substantial (4).

An extensive literature in behavioral economics examines gender gaps in measures such as competitiveness and risk tolerance (6, 7), but has devoted little attention to other important dimensions of heterogeneity such as race and ethnicity. As Table 1 shows, behavioral papers in economics routinely report the gender of participants but not their racial/ethnic background, both in global samples and in U.S.-based studies. Even when these data are collected, comparisons across racial and ethnic groups remain rare, with only a few notable exceptions (8, 9). This is noteworthy as this literature seeks to identify possible predictors of differences in life outcomes such as income, and a broader body of research emphasizing substantial within-country variation across socioeconomic and ancestral backgrounds (1, 4). A similar pattern appears in psychology, where “the vast majority of papers give no information about their sample apart from gender” (10).

**Table 1.** Proportion of studies with behavioral measures that report demographic information in leading economic journals (2020–2024)

Journal	Gender (all countries) (%)	Race/ethnicity (all countries) (%)	Gender (U.S. only) (%)	Race/ethnicity (U.S. only) (%)
Top five	66.4	18.5	69.6	39.1
Top field	66.3	5.4	52.4	14.3

Top five journals: *American Economic Review, Quarterly Journal of Economics, Journal of Political Economy, Econometrica,* and *Review of Economic Studies*; Top field journal: *Experimental Economics*.

The neglect of racial and ethnic variation in this type of research is particularly troubling in U.S. studies, where the population identifying as non-White has been steadily growing and now accounts for more than 40% of the population (11). Given the predominance of U.S. samples in behavioral research (1), failing to account for such variation risks producing results that systematically misrepresent large segments of the population. Influential experiments, for example, have shown that women are less competitive and less risk tolerant than men (6, 12)-findings that have become foundational in behavioral economics and related fields (13). However, as the race/ethnicity of participants is seldom reported, we do not know whether gender gaps in behavioral measures such as competitiveness and risk tolerance generalize across racial and ethnic lines. We also know little about how Whites compare to other racial and ethnic groups (8, 9). A major reason is that most behavioral studies draw on samples that offer little racial or ethnic diversity. In our review, 74% of studies relied either on convenience (student) samples-typically predominantly White-or on nationally representative samples that include too few minority participants to allow meaningful comparisons (*SI Appendix*). In effect, the “*W*” in WEIRD might as well stand for White.

Here, we analyze data that we collected from a large sample of U.S. adults (N=2,468), aged 25 to 54, stratified by race/ethnicity (using categories taken from the US Census) and gender, enabling well-powered comparisons across six race-ethnicity-gender (henceforth, REG) groups: Black women (N=424), Black men (N=402), Hispanic women (N=414), Hispanic men (N=407), (non-Hispanic) White women (N=409), and (non-Hispanic) White men (N=412). Our sampling strategy, together with poststratification weights, ensures that each REG group is nationally representative with respect to age, education, and the 2016 presidential vote, thereby strengthening the external validity of our findings.

We focus on competitiveness and risk tolerance-two behavioral measures widely studied in economics and the social sciences, which are considered central to shaping decision-making and known to exhibit robust gender differences (6, 12). Both are measured using incentivized tasks widely employed in the behavioral literature. Specifically, competitiveness is measured by whether individuals choose a competitive payment scheme over a fixed piece-rate scheme. Risk tolerance is measured by individuals’ choices from a standard menu of lotteries varying in risk exposure and payoffs. Further details are provided in *Methods* and *SI Appendix*.

## Results

**Fig. 1.**
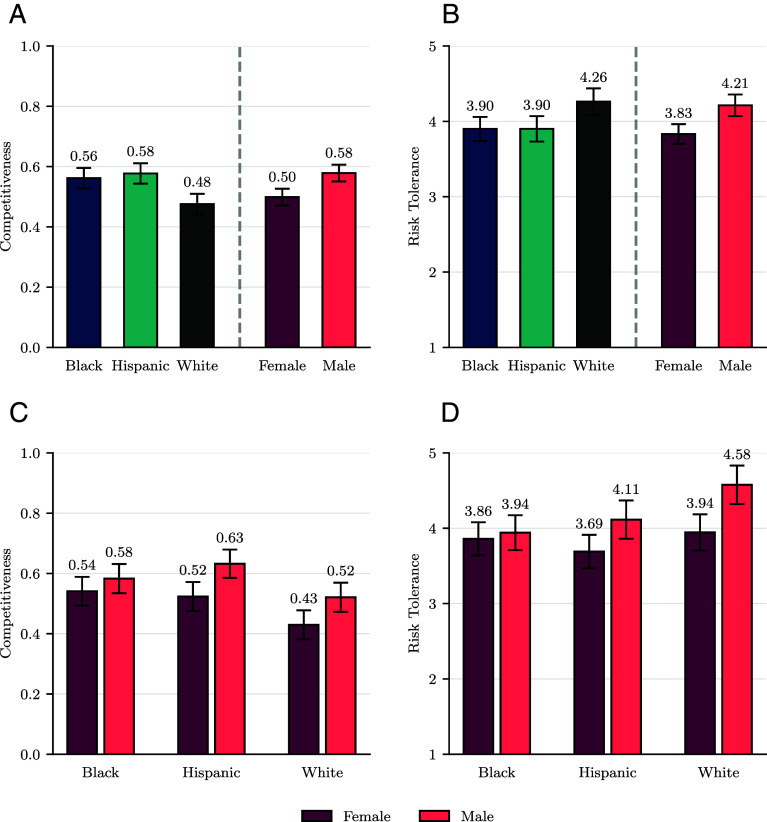
Competitiveness and risk tolerance, by race/ethnicity and gender (*A* and *B*) and their interaction (*C* and *D*). Whiskers show 95% CIs. Competitiveness is measured as the share of individuals selecting the competitive pay scheme in each group. Risk tolerance takes values between 1 (risk avoidance) and 9 (risk seeking), and a mean value of 4 indicates intermediate levels of risk tolerance (*SI Appendix*).

Fig. 1 summarizes our main findings concerning differences in competitiveness and risk tolerance across racial/ethnic/gender (REG) groups. Our first finding is that there exist pronounced differences between individuals that self-identify as Whites, on the one hand, and those who identify as Blacks/Hispanics on the other. Specifically, Panel (*A*) shows Blacks and Hispanics select the competitive payment scheme at a similar rate, 56% and 58% respectively (N=1,647, P=0.520, two-sided t test, as holds for all other statistical tests presented in this section). Whites, on the other hand, choose competition 47% of the time, i.e., 17.2% less often than Hispanics (N=1,642, P<0.001) and 14.3% less often than Blacks (N=1,647, P<0.001). Panel (*B*) shows a similar gap for risk tolerance: Blacks and Hispanics have an equal mean tolerance for risk (N=1,647, P=0.989), while Whites are 9.2% more risk tolerant on average than both Blacks (N=1,647, P=0.003) and Hispanics (N=1,642, P=0.004). These differences persist when controlling for education and income, suggesting that our socioeconomic status controls do not fully account for the observed racial and ethnic gaps.

How substantial are the differences between Whites and Blacks/Hispanics? One way to answer this is to compare the racial/ethnic gap in competitiveness and risk tolerance to the respective gender gap-the latter being the focus of much of behavioral research. By this metric, the racial/ethnic gaps are substantial. Specifically, as seen in Panel (*A*), men across racial/ethnic groups are 16.0% more likely to select the competitive payment scheme than females (N=2,467, P<0.001), in line with past findings in the literature. Thus, the overall gender gap in competitiveness is the same in magnitude to that between Whites and Blacks/Hispanics (16.0%). Similarly, Panel (*B*) reveals that men are 9.9% more tolerant of risk than women across RE groups (N=2,467, P<0.001). Again, this difference is similar to the 9.2% gap between Whites and Blacks/Hispanics mentioned above.

Our second result is that the gender gaps in competitiveness and risk tolerance do not generalize across races/ethnicities. Panel (*C*) shows that there is a sizable and statistically significant gender gap in the likelihood of choosing the competitive pay scheme for Hispanics (N=821, P=0.002) and Whites (N=821, P=0.009), but not for Blacks (N=826, P=0.226)—Black women do not shy away from competition compared to Black men. We reach the same conclusion for competitiveness when we control for other differences, such as risk tolerance and task performance (*Methods*), in a regression analysis. Panel (*D*) reveals a similar pattern for risk tolerance. Specifically, we find a gender gap for Hispanics (N=820, P=0.014) and Whites (N=820, P<0.001), but not for Blacks (N=825, P=0.616)—Black women are not less risk tolerant than Black men. As before, these findings are robust to controlling for individual income and education.

## Discussion

The data reveal that racial and ethnic differences in risk tolerance and competitiveness are substantial among U.S. adults, and comparable in magnitude to the widely studied gender gap. Not only do Whites—the most commonly studied racial group in behavioral science—differ from Blacks and Hispanics in terms of competitiveness and risk, but the gender gaps observed among Whites do not generalize to Blacks. These results raise fundamental questions about the generalizability of existing conclusions in the literature. These patterns are consistent with prior psychological research identifying differences in subjective risk perceptions between White men and other demographic groups, and linking them to social identity, cultural worldviews, and perceived control over outcomes (14, 15).

The pronounced racial and ethnic gaps in our study indicate that, to ensure the robustness and policy relevance of behavioral insights, research should examine variation across race and ethnicity. We have used the same legal-administrative race and ethnicity categories as the Office of Management and Budget and the US Census. These categories have conceptual limitations as they group together people with diverse ancestries and cultures, and rely on self-identification. Nevertheless, they play a central role in how U.S. institutions classify populations, monitor inequality, and allocate resources. To the extent that researchers use variations in behavioral measures to explain differences in life outcomes, studying behavioral differences along these categories is important.

Our findings show that relying on predominantly White samples while ignoring within-country racial and ethnic diversity creates challenges for generalization comparable to those posed by the field’s reliance on WEIRD samples.

matseccnt1

## Methods

The study was approved by the Institutional Review Board at NYU Abu Dhabi (046-2019) and administered by YouGov. Informed consent was obtained from all participants. We use the standard questions used by YouGov to elicit race/ethnicity (*SI Appendix*). Poststratification weights were calculated by YouGov and applied to ensure national representativeness across age, education, and political orientation within each gender and racial/ethnic group.

Competitiveness was measured using a variant of the design introduced by Niederle and Vesterlund (12). Participants first completed a piece-rate task involving the counting of 1’s in 4 × 4 matrices. In a second round, they performed the same task under a competitive payment scheme that rewarded only the top performer in a randomly paired dyad. In the third round, participants chose which payment scheme to repeat. Those opting for the competitive scheme are classified as competitive. Risk tolerance was measured using a standard incentivized lottery-choice task in which participants selected one option from nine binary lotteries that increased systematically in both expected payoff and risk.

## Supplementary Material

Appendix 01 (PDF)

## Data Availability

Deidentified experimental and survey data from human participants along with the replication code for this study are publicly available at the DataverseNL repository (DOI: 10.34894/JQNARD) (16).
