# Supplementary material for: The racial and ethnic gap in behavioral measures rivals the gender gap in the United States
Source: Proc Natl Acad Sci U S A. 2026 Feb 4;123(6):e2527671123. doi: 10.1073/pnas.2527671123 (PMC12891008; doi:10.1073/pnas.2527671123)
Supplement: Supplementary file 1 — Appendix 01 (PDF) [file pnas.2527671123.sapp.pdf]

PNAS

Supporting Information for

The racial and ethnic gap in behavioral measures rivals the gender gap in the United States

Aurélie Dariel,[1,2] John C. Ham,[2,3] Nikos Nikiforakis,[1,2,4] * Jan Stoop[5,6]

[1] Center for Behavioral Institutional Design, NYU Abu Dhabi, 129188 Abu Dhabi, UAE
[2] Division of Social Science, NYU Abu Dhabi, 129188 Abu Dhabi, UAE
[3] NYU Wagner School of Public Service, New York University, New York, NY 10012, USA
[4] Faculty of Arts and Science, New York University, New York, NY 10003, USA
[5] Erasmus School of Economics, Erasmus University Rotterdam, PO Box 1738, 3000 DR Rotterdam, the Netherlands
[6] Tilburg School of Economics and Management, Tilburg University, PO Box 90153, 5000 LE Tilburg, The Netherlands
* Corresponding author.

Email: nikos.nikiforakis@nyu.edu

This PDF file includes:

Literature Review Methodology
Sampling
Measuring behavioral traits and REG group membership

Literature Review Methodology
To explore the extent to which race and ethnicity are studied by behavioral economists, we undertook an extensive review of relevant research published in leading economics journals. We followed a multi-stage process to identify all relevant articles and had a team of independent coders answer questions about each article. Below, we provide details for each stage.

Stage 1. Article search
On February 12, 2025, we conducted a search in Web of Science, for articles published between January 1, 2020 and December 31, 2024. To keep the task manageable, we targeted six journals that have the reputation of publishing behavioral economic research of the highest quality. These journals are the "top-5" journals in economics (American Economic Review, Quarterly Journal of Economics, Journal of Political Economy, Econometrica, and Review of Economic Studies), and the top field journal (Experimental Economics). Specifically, we looked for articles in these journals that contained at least one of the following terms: behavioral, behavioural, experiment, experiments, experimentally, experimental, lab, laboratory, psychology, confidence, overconfidence, psychological, or competitiveness. This yielded 841 records.

Stage 2. Article screening for inclusion
We reviewed each of the 841 articles identified, and excluded those that did not involve data from human subjects, e.g., theoretical, historical, and simulation-based studies. After the screening, 584 papers were included in our review.

Stage 3. Coding
The 584 papers identified were randomly divided evenly into two subsets. Each subset was assigned to a pair of independent research assistants (RAs). Each RA independently reviewed and coded all papers in their assigned set. If a paper reported more than one distinct study or sample, it was duplicated into multiple rows and separately coded. The coding instrument included the following variables: Region and Country of Study, WEIRD Sample Indicator, Experiment Type (Lab, Field, Online, RCT, etc.), Testing of Formal Economic Theory, Measurement of Traits (e.g., risk, prosociality), Sample Type (student, general population, niche), Sampling Method, Demographic Reporting (race/ethnicity, gender, age, income, education, SES), Demographic analysis, and Compensation details. For each paper, when coders disagreed on any of the abovementioned variables, the discrepancy was resolved by a senior research assistant (predoctoral fellow), who conducted an independent review of the full text and issued a final decision.

Sampling
To obtain our data, we used the services of YouGov, an international research and analytics company with over 11 million panel members globally. Our online sample consists of 2,468 U.S. citizens, age 25–54, who participated in our study between January 13 and February 8 of 2021. The sample was balanced by gender and race/ethnicity. We consider six racial/ethnic and gender (REG) groups: Black men, Hispanic men, White men, Black women, Hispanic women, and White women. We use post-stratification weights to ensure that each REG group is nationally representative with regard to age, education, and the 2016 Presidential vote. Specifically, YouGov provided us with post-stratification weights based on the following variables: the 2016 presidential vote choice (Clinton, Trump, other), age (25-34, 35-44, 45-54), and education (up to high school degree, some college, undergraduate degree, postgraduate degree). The online study took approximately 20 minutes to complete. Respondents earned, on average, $8.80 from their participation, including a fee of $1.50 for showing up.

Measuring behavioral traits and REG group membership
Measuring competitiveness
Our measure of competitiveness is based on a task designed by Niederle and Vesterlund [2007]. The subjects face three tasks. In the first task, subjects are presented 4 X 4 matrices with 1's and 0's. Each subject has 75 seconds to count as many as possible 1's in a matrix. Subjects were paid 1 point for each correctly counted matrix. In Task 2, subjects again had 75 seconds to count as matrices as possible. Payment is 2 points per correctly counted matrix, but only when outperforming

a randomly matched participant. In Task 3, subjects could choose which task to redo: Task 1 or Task 2. Those choosing the competitive task are classified as competitors.
Specifically, subjects were given the following instructions:

- Task 1: In this task, you will be presented with a series of tables consisting of 1s and 0s, like the one pictured below. Your job is to count the number of 1s and enter your answer in the space provided. You will have 75 seconds to enter as many correct answers as possible. After 75 seconds, the task will end automatically and you will see your total score. There is no penalty for incorrect answers. If task this task is randomly selected for payment, you will be paid 1 point per correct answer. We call this the piece rate payment scheme.
  Click the arrow below when you are ready, and your 75 seconds will begin. Once you click the arrow below, you will not be able to interrupt the experiment until the task is completed. If you do, you will be disqualified from receiving any payments.
- Task 2: In this task, you will be given 75 seconds to correctly count the 1s in as many tables as possible. However, your earnings in this task depend on your performance relative to another participant in the experiment with whom you will be randomly matched. The participant will be drawn randomly from a representative sample of the US population.
  If task this task is randomly selected for payment, your payment will be determined as follows. If you solve more tables than the other participant, you will earn 2 points per correct answer. If the other participant solves more tables, you will earn 0 points for this task. If there is tie, the computer will randomly select one of you to receive 2 points per correct answer; the other will receive 0 points. We call this the tournament payment scheme.
  Click the arrow below when you are ready, and your 75 seconds will begin. Once you click the arrow below, you will not be able to interrupt the experiment until the task is completed. If you do, you will be disqualified from receiving any payments.
- Task 3: In this task, you will again be given 75 seconds to correctly count the 1s in as many tables as possible. Before this, however, you will have to choose which of the two previous payment schemes you prefer to determine your bonus earnings in case this task is selected for payment. More precisely, you will have to choose whether you prefer the Tournament scheme or the Piece Rate.
  The piece rate scheme will always pay 1 point per correct answer. The tournament scheme will pay 2 points per correct answer, if you are the one with the most correct answers (compared to another randomly drawn individual from the U.S. population). If your score is lower, you will be paid 0 points for this task. If there is a tie, the software will randomly select one of you to receive 2 points per correct answer; the other will receive 0 points. Which do you choose?
  - Piece Rate (1 point per correct answer)
  - Tournament (2 points per correct answer if you win)

Measuring risk attitudes

Risk attitudes were measured following the paradigm of Eckel and Grossman [2008]. Subjects were given a table with nine ranked lotteries. The least risky lottery is on top, and the most risky lottery is on the bottom. This variable therefore ranges from 1 to 9.
Below, we give the experimental instructions of this task:
In this task you are presented with 9 lotteries. Each lottery involves two different payments, both of which are equally likely to occur. The lotteries differ in the payments and their riskiness. Your task is to choose one of them. If this task is selected for payment, the outcome of the two associated with your chosen lottery that you will receive will be determined randomly by the computer. The 9 options you must choose from are:

	50 % Chance to receive	50 % Chance to receive
Lottery A	8	8
Lottery B	12	7
Lottery C	16	6
Lottery D	20	5
Lottery E	24	4
Lottery F	28	3
Lottery G	32	2
Lottery H	36	1
Lottery I	37	0

REG group membership
At the start of the study, we asked participants about their gender and race/ethnicity. Specifically, we asked them:

1) Are you?
- Male
- Female

2) What racial or ethnic group best describes you?
- White
- Black or African-American
- Hispanic or Latino
- Asian or Asian-American
- Native American
- Middle Eastern
- Two or more races
- Other

[The following question appeared if a participant selected an answer other than “Hispanic or Latino” in (2).]

3) (Are you of Spanish, Latino, or Hispanic origin or descent?
- Yes
- No

YouGov used the answers to (2) and (3) to distinguish between non-Hispanic White, non-Hispanic Black, and Hispanic, as part of the sampling process, resulting in a sample of 821 (non-Hispanic) Blacks, 826 Hispanic, and 821 (non-Hispanic) Whites.

SI References

1. Catherine C Eckel and Philip J Grossman. Forecasting risk attitudes: An experimental study using actual and forecast gamble choices. *Journal of Economic Behavior & Organization*,68(1):1–17, 2008.
2. Muriel Niederle and Lise Vesterlund. Do women shy away from competition? Do men compete too much? *The Quarterly Journal of Economics*, 122(3):1067–1101, 2007.
